# Artificial microRNA-derived resistance to Cassava brown streak disease

**DOI:** 10.1016/j.jviromet.2016.02.004

**Published:** 2016-05

**Authors:** Henry Wagaba, Basavaprabhu L. Patil, Settumba Mukasa, Titus Alicai, Claude M. Fauquet, Nigel J. Taylor

**Affiliations:** a National Crops Resources Research Institute, Namulonge, P.O. Box 7084, Kampala, Uganda; b Makerere University, P.O. Box 7062, University Rd, Kampala, Uganda; c ICAR-National Research Center on Plant Biotechnology, IARI, Pusa Campus, New Delhi 110012, India; d Centro Internacional de Agricultura Tropical, Cali, Apartado Aéreo 6713, Cali, Colombia; e Donald Danforth Plant Science Center, 975 North Warson Road, St. Louis, MO 63132, USA

**Keywords:** Cassava brown streak disease, *Cassava brown streak virus*, *Ugandan cassava brown streak virus*, Artificial microRNAs, Virus resistance

## Abstract

Artificial miRNAs (amiRNA) were generated targeting conserved sequences within the genomes of the two causal agents of Cassava brown streak disease (CBSD): *Cassava brown streak virus* (CBSV) and *Ugandan cassava brown streak virus* (UCBSV). Transient expression studies on ten amiRNAs targeting 21 nt conserved sequences of *P1*(CBSV and UCBSV), *P3*(CBSV and UCBSV), *CI*(UCBSV), *NIb*(CBSV and UCBSV), *CP*(UCBSV) and the un-translated region (3′-UTR) were tested in *Nicotiana benthamiana*. Four out of the ten amiRNAs expressed the corresponding amiRNA at high levels. Transgenic *N. benthamiana* plants were developed for the four amiRNAs targeting the *P1* and *NIb* genes of CBSV and the *P1* and *CP* genes of UCBSV and shown to accumulate miRNA products. Transgenic plants challenged with CBSV and UCBSV isolates showed resistance levels that ranged between ∼20–60% against CBSV and UCBSV and correlated with expression levels of the transgenically derived miRNAs. MicroRNAs targeting *P1* and *NIb* of CBSV showed protection against CBSV and UCBSV, while amiRNAs targeting the *P1* and *CP* of UCBSV showed protection against UCBSV but were less efficient against CBSV. These results indicate a potential application of amiRNAs for engineering resistance to CBSD-causing viruses in cassava.

## Highlights

• amiRNAs were produced for imparting resistance to Cassava brown streak disease.
• transgenic plants were produced expressing amiRNAs against CBSV and UCBSV.
• amiRNAs targeting conserved sequences of *P1*, *NIb* and CP genes were efficacious.
• levels of resistance to CBSD correlated with accumulation of detectable miRNA.

RNA silencing is a gene regulatory mechanism that controls transcript levels, either by suppressing transcription of messenger RNAs (transcriptional gene silencing [TGS]), or by activating a sequence-specific RNA degradation process (post-transcriptional gene silencing [PTGS]) (Baulcombe, 2004, Baulcombe, 2005). RNA silencing mechanisms involve small RNAs (sRNAs) that range in size between 21–24 nt, classified majorly into microRNAs (miRNAs) and small interfering RNAs (siRNAs) (Axtell, 2013). While miRNAs are generated from imperfect fold-back regions of long endogenous primary transcripts called pri-miRNAs (Bartel, 2004, Brodersen and Voinnet, 2009, Mallory and Vaucheret, 2006), siRNAs are processed from long double stranded RNAs (dsRNAs). MiRNA biogenesis begins with a cut at the base of their stem-loop structures, leading to formation of precursors (pre-miRNAs), that are further processed to produce ∼21 nt miRNA duplexes using DCL1 (Voinnet, 2009). SiRNAs on the other hand, are produced via DCL4 cleavage of perfectly base paired dsRNAs.

Engineering plants for resistance to viruses has been achieved in many species via homology-dependent RNA silencing by transgenic expression of viral sequences designed to produce siRNAs and trigger the RNA silencing pathway (Prins et al., 2008). More recently, expression of artificial microRNAs (amiRNAs) has been reported to result in resistance to plant virus pathogens (Duan et al., 2008, Fahim et al., 2012, Kung et al., 2012, Niu et al., 2006, Zhang et al., 2011). The latter is achieved by mimicking the intact secondary structure of endogenous miRNA precursors using oligo nucleotide substitutions to produce targeted silencing of desired viral genes (Ai et al., 2011, Niu et al., 2006, Qu et al., 2012). The use of short sequences via amiRNAs is considered to be advantageous over long hairpin-mediated silencing due to reduced off-target effects on host genes. In addition, such sequences also circumvent concerns that transgenically expressed, long viral sequences may complement or recombine with non-target viruses when used for agricultural applications. Cassava brown streak disease (CBSD) is caused by *Ugandan cassava brown streak virus* (UCBSV) and *Cassava brown streak virus* (CBSV); family *Potyviridae*, genus *Ipomovirus,* and poses a major threat to cassava production in East and Central Africa (Legg et al., 2014). CBSD is transmitted by the whitefly *Bemisia tabaci* (Gennadius) (Maruthi et al., 2014, Maruthi et al., 2005) and disseminated by planting of infected cuttings. Its widespread occurrence in East Africa has become a major constraint to cassava production in the region, resulting in 30–85% yield reductions in farmers’ fields (Pennisi, 2010). Transgenic control of CBSD in cassava plants by expression of ∼900 nt hairpin construct derived from the coat protein of UCBSV was recently demonstrated under greenhouse conditions and field conditions (Odipio et al., 2014, Ogwok et al., 2012, Patil et al., 2011, Yadav et al., 2011). The importance of this disease, however, requires continued efforts to develop additional effective resistance strategies.

In the present study, the efficacy of an amiRNA-based strategy to control CBSD was explored in *N. benthamiana.* Virus sequences for targeting by amiRNAs were identified using Clustal W (Lasergene Version 8) by aligning NCBI accessions of CBSV and UCBSV, namely GQ329864, GU563327, FN434437, FN434436, GQ169761, GQ169760, GQ169759, GQ169758, FJ185044, FJ039520, FN434109 and HM181930. Conserved oligo sequences of ∼21 nt in size were identified from the *P1*, *P3*, *CI*, *NIb* and *CP* genes and 3′-UTR of CBSV and UCBSV (Supplementary Table 1). Sequences with a 5′-A were preferentially selected to match the sequence requirements of amiRNAs, as previously described by Niu et al. (2006). Oligonucleotide-directed mutagenesis was then employed to incorporate sequences for the desired 21 nt insertions within the mature miR159a precursor from *A. thaliana,* using primers shown in Supplementary Table 1. PCR-amplified DNA fragments of the amiRNA precursor were subsequently ligated into shuttle vector CGT11003 (Patil et al., 2011) downstream of the constitutive *Cassava vein mosaic virus* (CsVMV) promoter (Verdaguer et al., 1998). The resulting amiRNAs were cloned into the binary vector pCambia2300 (Acc. No. AF234315) to generate pre-amiRNAs and mobilized into *Agrobacterium tumefaciens* strain GV3101 for transient studies, and strain LBA4404 for stable transformation of *N. benthamiana*.

Initially, *Agrobacteria* harboring amiRNA cassettes were transiently expressed via agro-infiltration into 21-day-old *N. benthamiana* plants grown in a growth chamber as described previously (Ogwok et al., 2010, Patil et al., 2011). Each treatment consisted of nine plants and the experiment performed at least three times. For all agro-infiltration experiments, the near full-length coat protein (ΔFL-UCBSV CP) RNAi construct was included as a positive control in each experiment (Patil et al., 2011) to assess relative expression levels of the small RNAs. amiRNAs expressed by each construct was determined by Northern blot analysis at three days after agro-infiltration using a mixture of 3′-end-labeled DNA oligos (Roche Applied Science, Indianapolis, IN, USA), complementary to the expressed amiRNAs used as the probe.

**Fig. 1:**
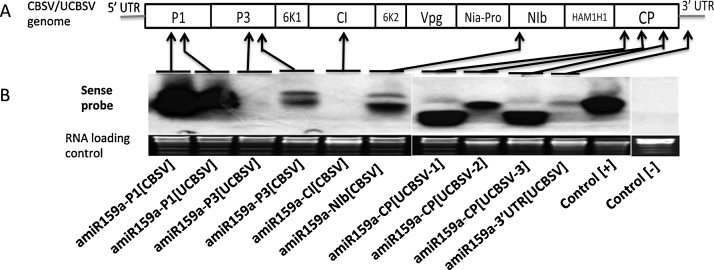
Transient expression of artificial microRNAs designed from genomic sequences of *Cassava brown streak virus* (CBSV) and *Ugandan cassava brown streak virus* (UCBSV). (A) Genome structure of CBSV and UCBSV. *P1*: Proteinase 1, *P3*: third protein, *CI*: cytoplasmic inclusion protein, *NIb*: replicase protein, *CP*: Coat protein. (B) Northern analysis showing expressed amiRNAs designed from conserved sequences of CBSV and UCBSV at three days after *Agrobacterium*-infiltration in tobacco leaves. Control [+] indicates transiently expressed short interference RNAs with ΔFL-CP[UCBSV] (p718) hairpin of the coat protein sequence (Patil et al., 2011). Control [−] represents plants *Agro-*infiltrated with the empty pCambia2300 vector.

Expression studies showed that microRNAs of 21 nt in length were detectable from leaf tissues agro-infiltrated with constructs amiR159a-P1[CBSV], amiR159a-P1[UCBSV], amiR159a-NIb[CBSV], amiR159a-CP[UCBSV-1], amiR159a-CP[UCBSV-2] and amiR159a-CP[UCBSV-3] using a mixture of sense probes targeting each of the amiRNAs (Fig. 1B). Constructs amiR159a-P3[UCBSV], amiR159a-CI[CBSV] and amiR159a-3′UTR[UCBSV] showed little or no expression of miRNAs. Interestingly, miRNAs expressed by amiR159a-CP[UCBSV-1] and amiR159a-CP[UCBSV-3] were smaller in size than the expected 21 nt (Fig. 1B). Accumulation of undersized (<21 nt) miRNAs from amiRNA precursors has been reported previously in transgenic petunia (Guo et al., 2014). As a result, these amiRNA constructs were not used to develop transgenic plants. Constructs amiR159a-P3[UCBSV] and amiR159a-NIb[CBSV] accumulated miRNAs at very low levels. No detectable accumulation of miRNAs was observed from amiRNA constructs amiR159a-CI[CBSV] and amiR159a-3′UTR[UCBSV] (Fig. 1B).

**Fig. 2:**
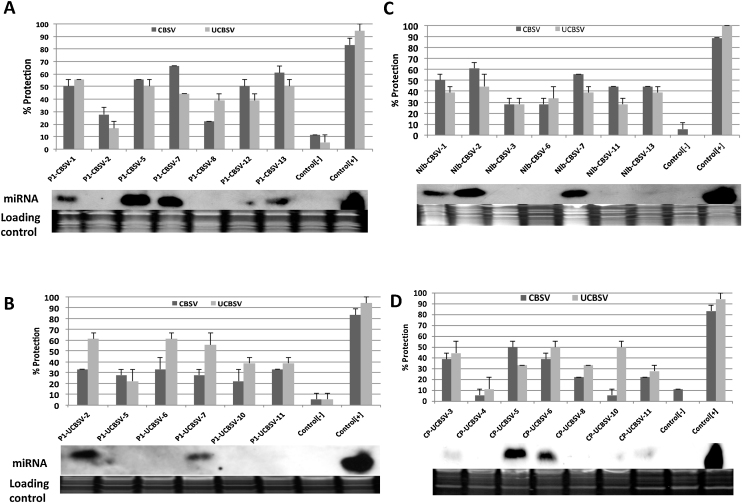
Percentage protection and expression levels of amiRNA in transgenic *T*_1_*N. benthamiana* plants. Greenhouse-grown plants confirmed to be transgenic using the neomycin phosphotransferase protein (NPTII) were sap-inoculated after planting with CBSV-[TZ:Nal:07] and UCBSV-[UG:Nam:04] isolated from CBSD-infected cassava plants at 21 days of age. Control [+] represents plants from line FL17 harboring the ΔFL-UCBSV CP hairpin siRNA construct (Patil et al., 2011). Control [−] represents transgenic plants harboring the plasmid pCambia2300 (empty vector) challenged with CBSV and UCBSV. Average values were calculated from three independent challenge experiments consisting of nine plants each. Percent protection indicates the average number of plants showing no symptoms of CBSV and UCBSV infection in *N. benthamiana* out of total experimental plants. Error bars shown are the standard errors (SE) of the mean. Expression levels of amiRNAs as determined by Northern blot analysis are shown below each of the challenged lines. (A) *P1*(CBSV) transgenic lines, (B) *P1*(UCBSV) transgenic lines, (C) *NIb*(CBSV) transgenic lines and (D) *CP*(UCBSV) transgenic lines. Northern analysis was performed on a sample of each transgenic line before challenge with CBSV or UCBSV.

amiRNA constructs targeting *P1* and *NIb* genes (CBSV) and *P1* and *CP* genes (UCBSV) (Table 1), which were shown to accumulate significant levels of miRNA from the transient expression assays, were used to produce stable transgenic plants of *N. benthamiana* (Horsch et al., 1985) in addition to the empty vector control. The previously generated tobacco transgenic line FL-17, derived from the coat protein RNAi hairpin construct p718 ΔFL-UCBSV (Patil et al., 2011), was included as a positive control. Seven hemizygous *T*_1_ lines were sap-inoculated at 21 days after transfer to soil (Patil et al., 2011) with virus isolates CBSV-[TZ:Nal:07] and UCBSV-[UG:Nam:04] to determine the level of resistance against CBSV and UCBSV respectively. Furthermore, seeds from *T*_1_ segregants were selected on kanamycin antibiotic media to obtain homozygous *T*_2_ transgenic lines. *T*_2_ plants were also challenged with CBSV and UCBSV in a manner similar to that of the *T*_1_ lines. Results showed that 90–100% of the non-transgenic and empty vector control plants developed typical CBSV and UCBSV symptoms at 4–10 days after sap inoculation and did not recover from disease over the 30-day observation period. Of plants expressing siRNAs from line FL-17 transgenic for siRNA construct p718, 80–100% remained disease-free (80–100% resistance) in a manner similar to that reported previously (Patil et al., 2011). amiRNA constructs targeting *P1*(CBSV) and *NIb*(CBSV) showed 25–65% resistance against CBSV across the seven *T*_1_ transgenic lines tested, with the four lines targeting *P1*(CBSV) and the three lines targeting *NIb*(CBSV) showing ≥50% protection against this CBSD causal agent (Fig. 2A and C). Plants transgenic for amiRNA constructs *P1*(CBSV) and *NIb*(CBSV) were also resistant against challenge with UCBSV at levels comparable to those against CBSV (Fig. 2A and C). This cross-protection was observed despite 4 and 3 nt mismatches present in these amiRNAs (Table 1). Three plant lines out of the seven tested for constructs designed to target *P1* and *CP* of UCBSV displayed ≥50% resistance against challenge with UCBSV. However, the UCBSV-derived constructs were less effective at imparting resistance against CBSV, with only one transgenic line (line 5) showing ≥50% resistance to this pathogen (Fig. 2B and D). A comparison of all four amiRNA constructs tested shows that amiRNAs targeting *P1*(CBSV) had more amiRNA expressing lines conferring resistance against CBSV and UCBSV, with three out of four lines tested showing ≥50% resistance against both pathogens. None of the other constructs tested had lines expressing amiRNAs with resistance ≥50% against both pathogens.

Northern blot analysis was performed to assess levels of miRNA accumulation within the transgenic plant lines using 3′-end labeled oligo probes specific to each of the constructs. amiRNAs were detected in the transgenic plants of all four amiRNA constructs. Using Image J ver 1.46 program (Schneider et al., 2012), the intensity of expression of amiRNAs was measured using an “oval” elliptical selection tool and statistically correlated to the level of resistance against each line against CBSV and UCBSV. The amiRNA signal intensity in some of the lines was comparable to that of the siRNAs derived from the control F-17 p718 line using the Image J software, measured over a uniform area (Fig. 2A–D). Plant lines that displayed high levels of resistance to CBSD also showed high amiRNA accumulation. The correlation coefficient between expression and resistance was generally positive, ranging between 0.70–0.80 for CBSV and 0.59–0.80 against UCBSV. Visually, some lines displayed very low expression of miRNAs but had some low level protection against both viruses. Interestingly, the level of protection obtained in these lines was still higher than the level of protection obtained against the empty plasmid vector line. Positive correlations between expression levels of miRNAs and resistance to virus challenge have also been reported against *Cucumber mosaic virus* (CMV) and *Potato virus Y* (PVY) and *Potato virus X* (PVX) amiRNA-mediated resistance (Ai et al., 2011, Qu et al., 2007).

**Fig. 3:**
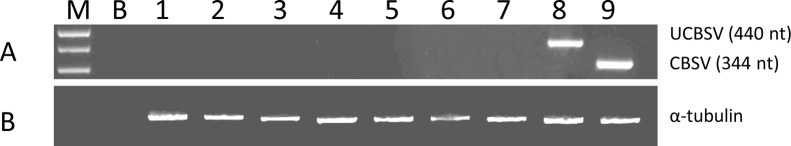
Reverse transcriptase-polymerase chain reaction (RT-PCR) for simultaneous detection of *Ugandan cassava brown streak virus* and *Cassava brown streak virus*. The primers CBSDDF2 and CBSDDR were used to amplify 440 and 344 nt of UCBSV- and CBSV-(TZ:Nal:07) viruses simultaneously from cDNA obtained from total RNA extracts of both asymptomatic samples. (A) RT-PCR of samples obtained from transgenic sap-inoculated asymptomatic and symptomatic plants. M—marker; B—PCR blank while samples 2–7 are asymptomatic transgenic plants. Samples 8 and 9 were symptomatic plants infected with UCBSV and CBSV respectively. Samples 1–4 were sap-inoculated with CBSV and 5–6 sap-inoculated with UCBSV. (B) RT-PCR of the same samples using the Tobefs and TobefA primers that amplify the constitutively expressed α-tubulin control gene.

Thirty days after sap inoculation, plants were analyzed by RT-PCR to determine presence of CBSV and UCBSV using primers that simultaneously amplify both viruses (Mbanzibwa et al., 2011a). Symptomatic wild type and transgenic plants were found to be positive for presence of CBSV and UCBSV, while asymptomatic plants were free of detectable virus (Fig. 3). This indicated that no systemic accumulation of CBSV or UCBSV occurred within asymptomatic plants over the observation period.

In the present study, resistance to CBSV and UCBSV in transgenic plants was observed in some lines that showed no accumulation of detectable miRNAs, for example, line P1-CBSV-2 and line P1-CBSV-8. There are two plausible explanations for this observation: (1) the level of expression of some amiRNAs was below that detectable by Northern blot analysis; and (2) a mechanism possibly mediated by non-cleavage of the target is operational. While cleavage is the most predominant mechanism of action in plants (Brodersen and Voinnet, 2009), recent reports also suggest that translational repression is also common (Li et al., 2013, Ma et al., 2013). In *A. thaliana,* Li et al. (2014) reported that *MYB33*, a gene regulated by miR159a microRNA, was regulated via a non-cleavage mechanism. Methylation of RNA as a means of regulating gene expression has also been reported by Fu et al. (2014) and reviewed by Motorin and Helm (2011). Whether one or both of these mechanisms may be functioning in the present study requires further investigation.

CBSV and UCBSV have a similar genome structure (Fig. 1) and share approximately 70% nucleotide sequence identity (Mbanzibwa et al., 2011b, Ndunguru et al., 2015, Winter et al., 2010). The *CP* is the most conserved, while *Ham1* is the least conserved between CBSV and UCBSV isolates examined to date (Winter et al., 2010). Plant miRNAs require perfect homology at positions 3, 6, 9 and 12 for effective silencing (Fahim and Larkin, 2013). Previously, Schwab et al. (2005) formulated empirical parameters that govern miRNA target prediction that included no mismatches in the “seed region” (positions 2–12 of the 5’end of the miRNA) and no mismatch at cleavage positions 10 and 11. These parameters were adhered to as closely as possible when designing the amiRNAs employed in this study. However, Clustal analysis revealed very few conserved sequences in *P1, NIb, CP* and 3′-UTR of CBSV and UCBSV that conformed to miRNA design requirements (Ossowski et al., 2008). This absence of conservation (Table 1) may explain why 100% protection against the CBSD viral agents was not observed, with maximum levels of resistance obtained in the range of 50–60%. The low resistance obtained with amiRNAs could also be due to inoculations performed with imperfectly matching CBSV and UCBSV isolates. In mismatched miRNAs, RISC is not recycled adequately, making the silencing process more energy demanding and subsequently lowering efficiency of cleavage (Li et al., 2014). Interestingly, in previous studies using the ΔFL-UCBSV CP hairpin RNAi construct, Patil et al. (2011) found that mismatches were acceptable in siRNA-mediated resistance against CBSV and UCBSV. This phenomenon may explain the dual protection observed for some of the amiRNA constructs targeting *P1* and *NIb* genes of CBSV reported here (Fig. 2A-D and Supporting Information Fig. 1A–D).

Previous investigations utilizing amiRNAs for resistance to plant viruses have targeted suppressors of RNA silencing, resulting in reports of 100% protection against the pathogen (Ai et al., 2011, Niu et al., 2006). The present study targeted the *P1* genes of CBSV and UCBSV, also known to be suppressors of RNA silencing (Mbanzibwa et al., 2009). However, whereas targeting the *P1* of CBSV provided good resistance against both CBSV and UCBSV, targeting the *P1* gene of UCBSV was less efficient against CBSV. The reason for this is not understood, but CBSV is considered to be a more aggressive disease agent compared to UCBSV (Winter et al., 2010). amiRNAs targeting the *CP* provided strong resistance against Grapevine fanleaf virus in Grapevines (Jelly et al., 2012). In the present investigations, targeting the *CP* sequence was less effective compared to the *P1* gene for imparting resistance to both CBSV and UCBSV. Additionally, transgenic tomato plants expressing amiR-AV1-1 targeting the middle region of the AV1 transcript against *Tomato leaf curl New Delhi virus* (ToLCNDV) were tolerant to the virus (Peng et al., 2014). Here, we targeted the *CP* and *NIb* gene and have obtained elevated resistance against CBSV and UCBSV (Fig. 2A and B). Targeting the *P1*[CBSV] and *NIb*[CBSV] was more efficient against CBSV and UCBSV compared to *CP*[UCBSV] and *P1*[UCBSV] (Fig. 2A and B).

Levels of disease resistance obtained by expressing amiRNAs in this study were generally 50% lower than those previously reported through expression of the ΔFL-UCBSV CP hairpin siRNA construct (Patil et al., 2011). This was possibly due to the smaller target sequences (21 nt) of the amiRNAs compared to the ∼900 nucleotides employed within the siRNA hairpin construct (Patil et al., 2011). *In silico* analysis of the possible siRNAs that arise from the CP hairpin (p718) construct including 1 or 2 mismatches showed different possible combinations of siRNAs against CBSV and UCBSV (Patil et al., 2011). Since infections with CBSV and/or UCBSV are common in cassava fields (Mbanzibwa et al., 2011a), durable resistance against both viruses will most likely require the combination of different amiRNAs designed in polycistronic manner to target multiple conserved genes. This is an approach similar to that reported for wheat by Fahim et al. (2012).

In conclusion, we report that expression of amiRNA targeting conserved genes in the CBSV and UCBSV genomes results in resistance against these viruses. These results add another potential source of resistance against CBSD-causing viruses in cassava. Future studies will focus on stacking amiRNA expression cassettes such as *PI* and *Nib* for co-expression within the same plant and investigating the efficacy of this approach within transgenically modified cassava.

## Conflict of interest

The authors have no conflict of interest to declare.

## Figures and Tables

**Table 1:** Conservation in four selected amiRNA targets derived from the *Cassava brown streak virus* (CBSV) and *Ugandan cassava brown streak virus* (UCBSV) genomes. (A) Alignment of five published genomes of CBSV available at NCBI. (B) Alignment of eight UCBSV sequences available at NCBI with target sequences. Mismatched nucleotides are indicated in red. Isolates indicated in bold were used to challenge transgenic *N. benthamiana* plants.
