# Supplementary figures and images for: Artificial microRNA-derived resistance to Cassava brown streak disease
Source: J Virol Methods. 2016 May;231:38–43. doi: 10.1016/j.jviromet.2016.02.004 (PMC4819903; doi:10.1016/j.jviromet.2016.02.004)

## Slide 1
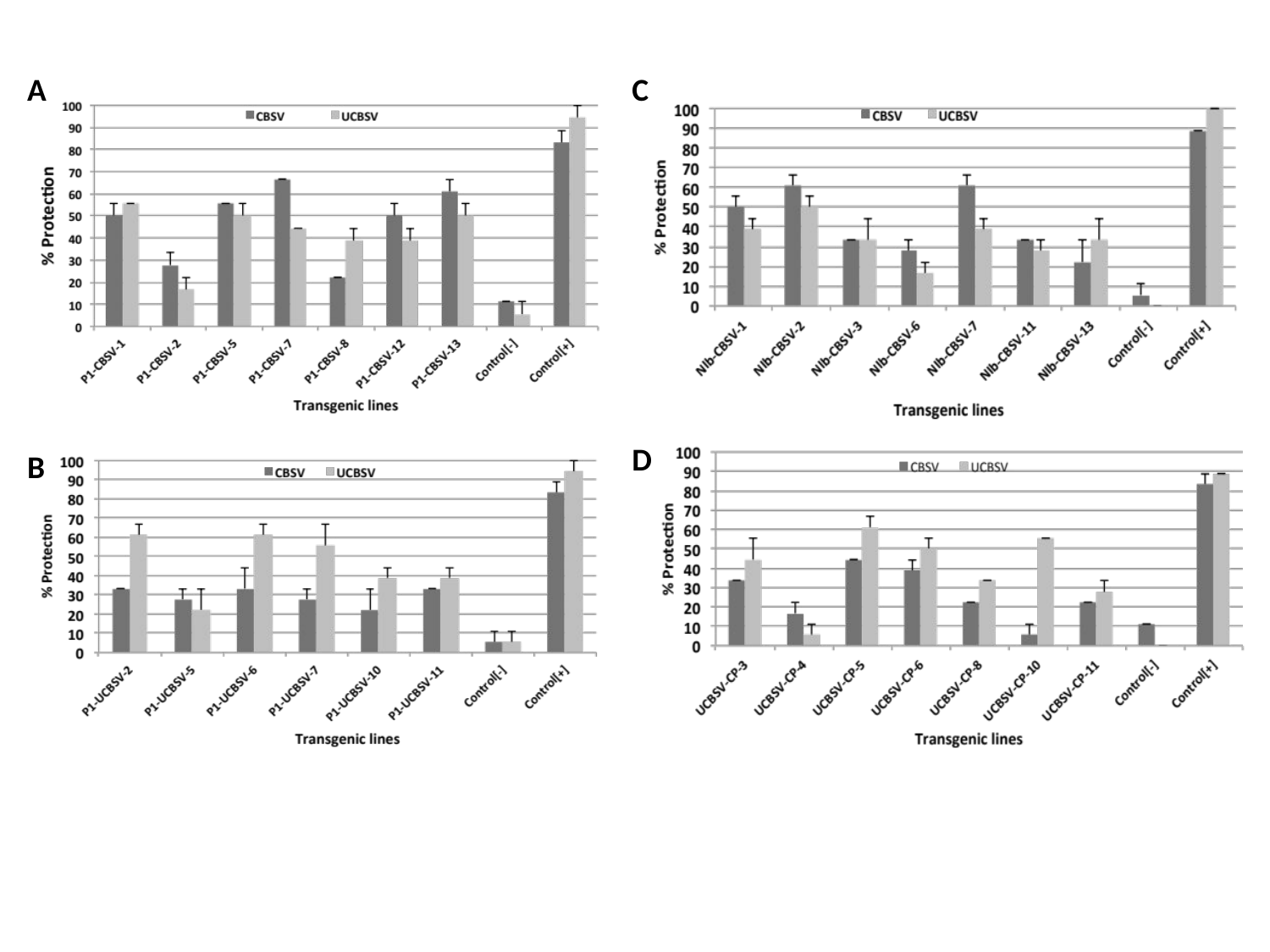

A
C
D
B

Supplement: Supplementary file 1 [file mmc1.ppt]
