# Supplementary material for: Artificial microRNA-derived resistance to Cassava brown streak disease
Source: J Virol Methods. 2016 May;231:38–43. doi: 10.1016/j.jviromet.2016.02.004 (PMC4819903; doi:10.1016/j.jviromet.2016.02.004)
Supplement: Supplementary file 2 [file mmc2.doc]

| **Construct name** | **Primer sequence (5’–3’)** |
| --- | --- |
| amiR159a-P1[CBSV] | F: TCA*TCTAGA*TGATCTGACGATGGAAGATGGTGAGACCTGGTTGGAGTCATGAGTTGAGCAGGGTAAAG  R: CATTTCGAAATGATGATGGTGAGACCTGGTTGGAGTAAGAGTAAAAGCCATTAAAG |
| amiR159a-P1[UCBSV] | F: TCATCTAGATGATCTGACGATGGAAGAAGAGTTGATTAGATTTGGTTCATGAGTTGAGCAGGGTAAAG  R: CAT*TTCGAA*ATGATGATTCAGAGTTGATTAGATTTGGTTGAGTAAAAGCCATTAAAG |
| amiR159a -P3[UCBSV] | F: TCA*TCTAGA*TGATCTGACGATGGAAGTTAGAAGCACAAGAAATTATGCATGAGTTGAGCAGGGTAAAG  R: CAT*TTCGAA*ATGTTAGAAGCACAAGAAATTATGGAAGAGTAAAAGCCATTAAAG |
| amiR159a-P3[CBSV] | F: TCA*GGTACC*TGATCTGACGATGGAAGTTCTTGTGG*TCTAGA*ATAGTTCATGAGTTGAGCAGGGTAAAG  R: CAT*TTCGAA*ATGTTCTTGTGGTCTAGAATAGTTGAAGAGTAAAAGCCATTAAAG |
| amiR159a-CI[CBSV] | F: TCA*TCTAGA*TGATCTGACGATGGAAGGAAGCAAATTATATATTCACACATGAGTTGAGCAGGGTAAAG  R: CAT*TTCGAA*ATGGAAGCAAATTATATATTCACAGAAGAGTAAAAGCCATTAAAG |
| amiR159a-NIb[CBSV] | F: TCA*TCTAGA*TGATCTGACGATGGAAGGATTGTGATGGTAGTAGGTTTCATGAGTTGAGCAGGGTAAAG  R: CATTTCGAAATGGATTGTGATGGTAGTAGGTTTGAAGAGTAAAGCCATTAAAG |
| amiR159a-CP[UCBSV-1] | F: TCA*TCTAGA*TGATCTGACGATGGAAGAAACAAAGAAGAGGCCATGTGCATGAGTTGAGCAGGGTAAAG  R: CAT*TTCGAA*ATGAAACAAAGAAGAGGCCATGTGGAAGAGTAAAAGCCATTAAAG |
| amiR159a-CP[UCBSV-2] | F: TCA*TCTAGA*TGATCTGACGATGGAAGTGCTTCAGGAGTTGAAGTTGACATGAGTTGAGCAGGGTAAAG  R: CAT*TTCGAA*ATGTGCTTCAGGAGTTGAAGTTGAGAAGAGTAAAAGCCATTAAAG |
| amiR159a-CP[UCBSV-3] | F: TCA*TCTAGA*TGATCTGACGATGGAAGCTGGCAGCGAATGTTGGTAGACATGAGTTGAGCAGGGTAAAG  R: CAT*TTCGAA*ATGCTGGCAGCTAATGTTGGTAGAGAAGAGTAAAAGCCATTAAAG |
| amiR159a-3’UTR[UCBSV] | F: TCA*TCTAGA*TGATCTGACGATGGAAGGGAGATCTTTCTCCATATCCTCATGAGTTGAGCAGGGTAAAG  R: CAT*TTCGAA*ATGGGAGATCTTTCTCCATATCCTGAAGAGTAAAAGCCATTAAAG |

**Supp. Table 1**. Primers and primer sequences used to construct amiRNA constructs, specifying conserved regions of *Cassava brown streak virus* (CBSV) and *Ugandan cassava brown streak virus* (UCBSV). Forward primers contain XbaI site (shown in italics) except amiR159a-P3[UCBSV] which contains a KpnI site. Reverse primers (amiRNA*) have a Bst*B*I site (shown in italics). The selected conserved sequences are underlined.
